# Supplementary material for: Global Profiling of Protein β-hydroxybutyrylome in Porcine Liver
Source: Biology (Basel). 2025 Sep 2;14(9):1183. doi: 10.3390/biology14091183 (PMC12467457; doi:10.3390/biology14091183)
Supplement: Supplementary file 1 [file biology-14-01183-s001.zip › Sup Figs.pdf]

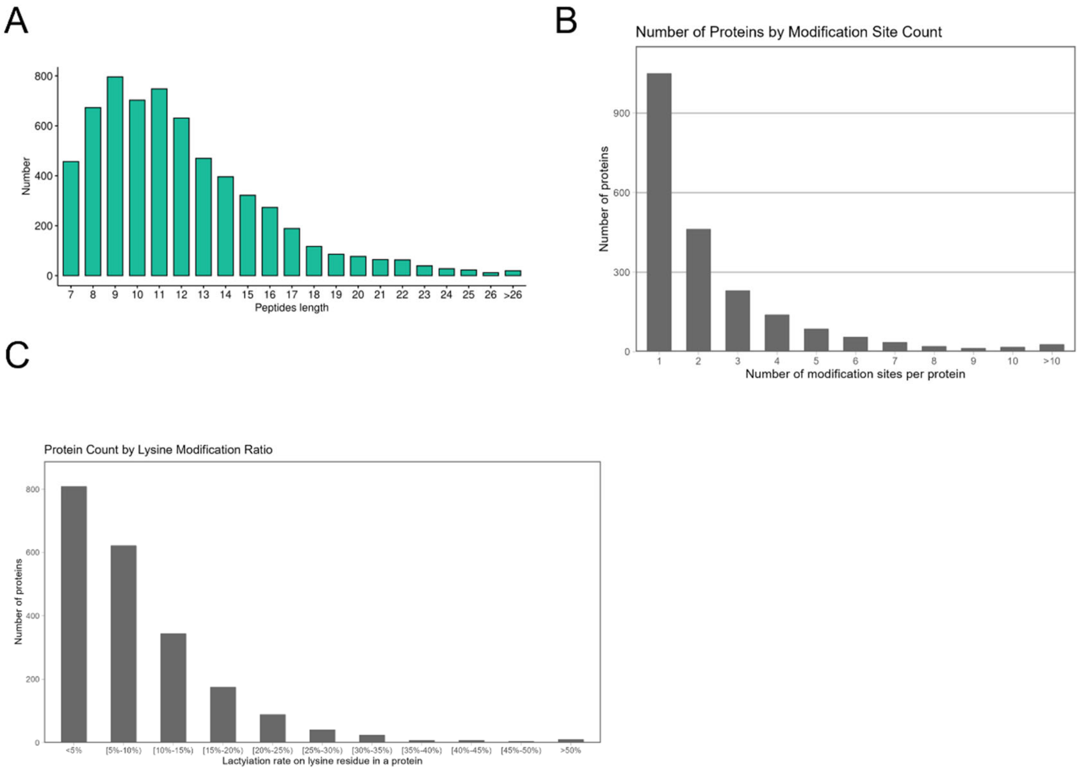

Supplementary Figure S1. Quality control of proteomics. A. The distribution pattern of peptide segment lengths. The abscissa represents the number of amino acids contained in the peptide segments, and the ordinate represents the number of peptide segments. B. The number of sites where proteins undergo modifications. The abscissa represents the number of lysines that undergo modifications on the same protein, and the ordinate represents the number of proteins. C. The proportion of lysines that undergo modifications in the same protein among all lysines.

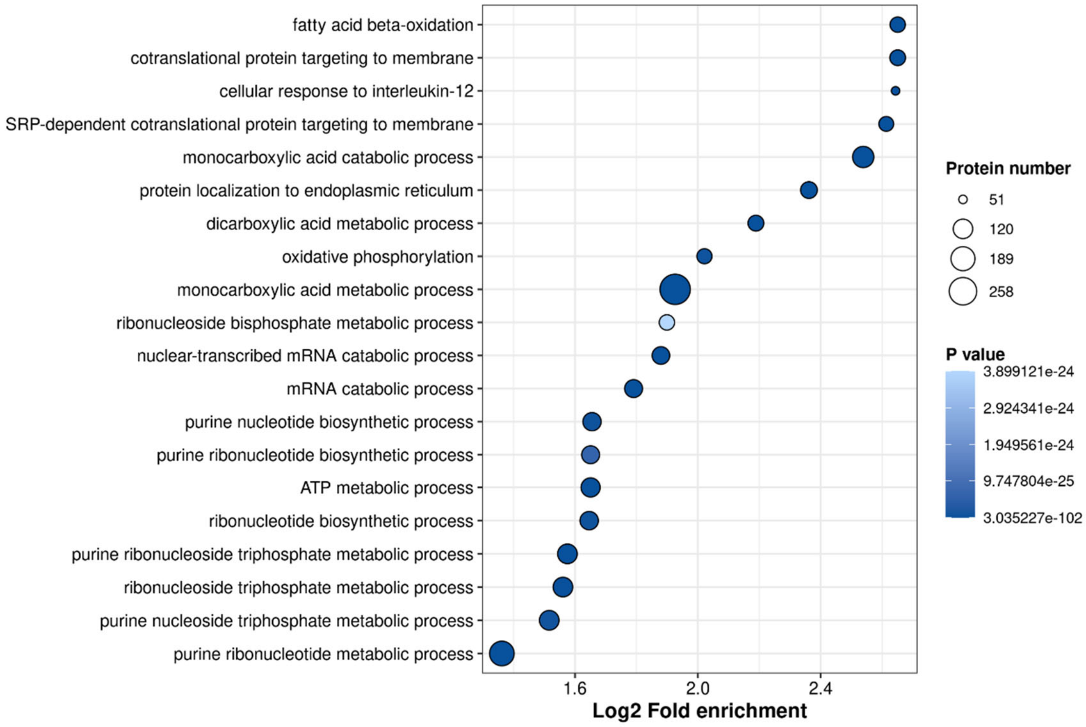

Supplementary Figure S2. GO enrichment of all Kbhb proteins.

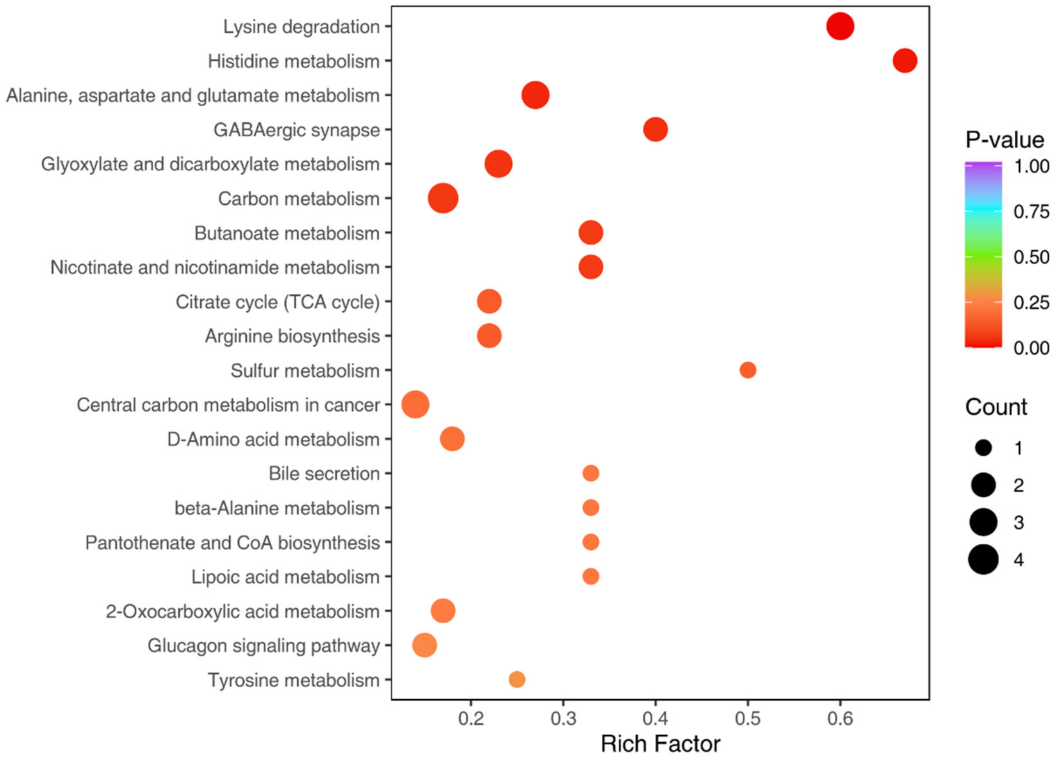

Supplementary Figure S3. Protein domain enrichment of all Kbhb proteins.

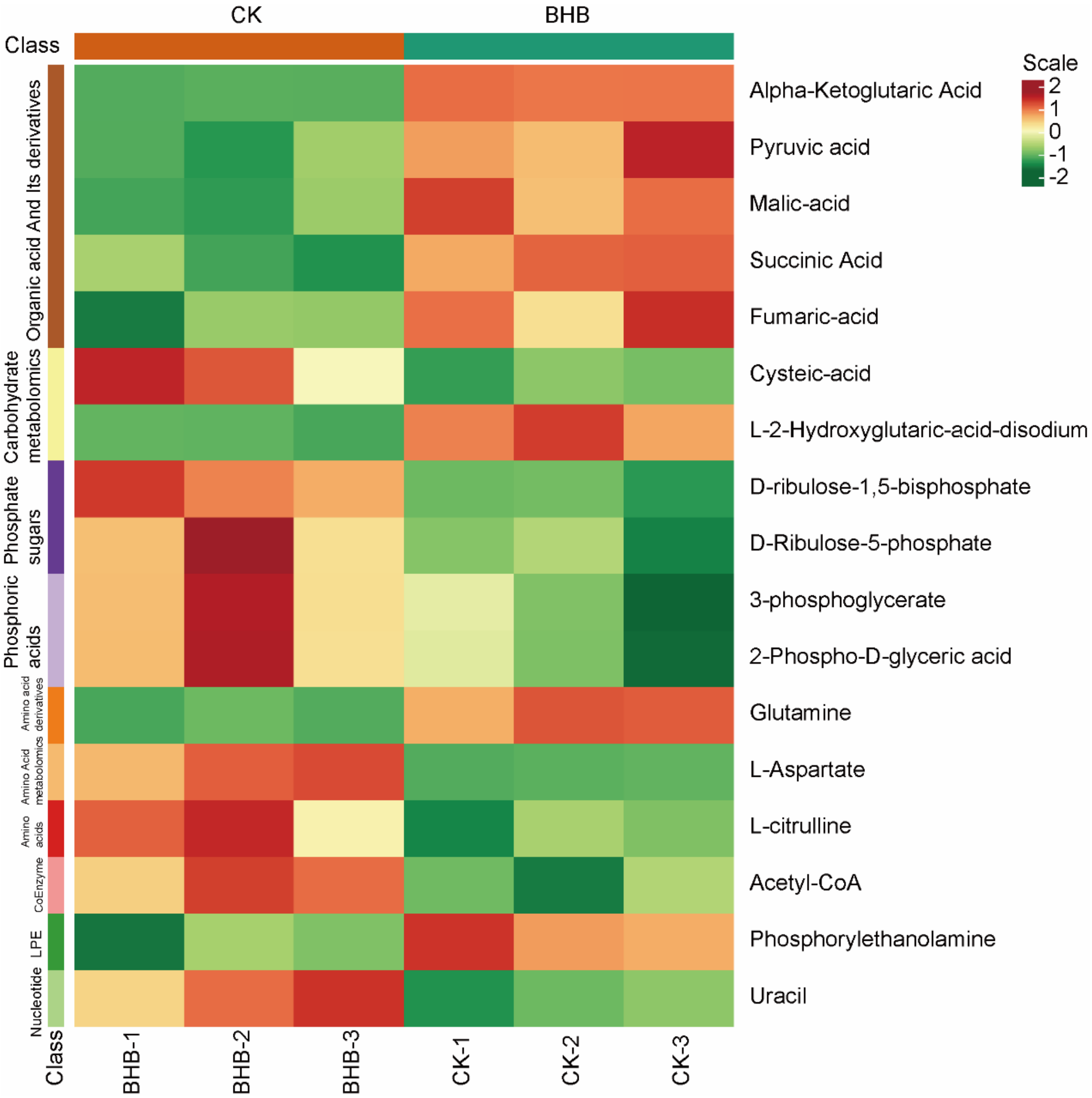

Supplementary Figure S4. All the different metabolites before and after the addition of BHB.

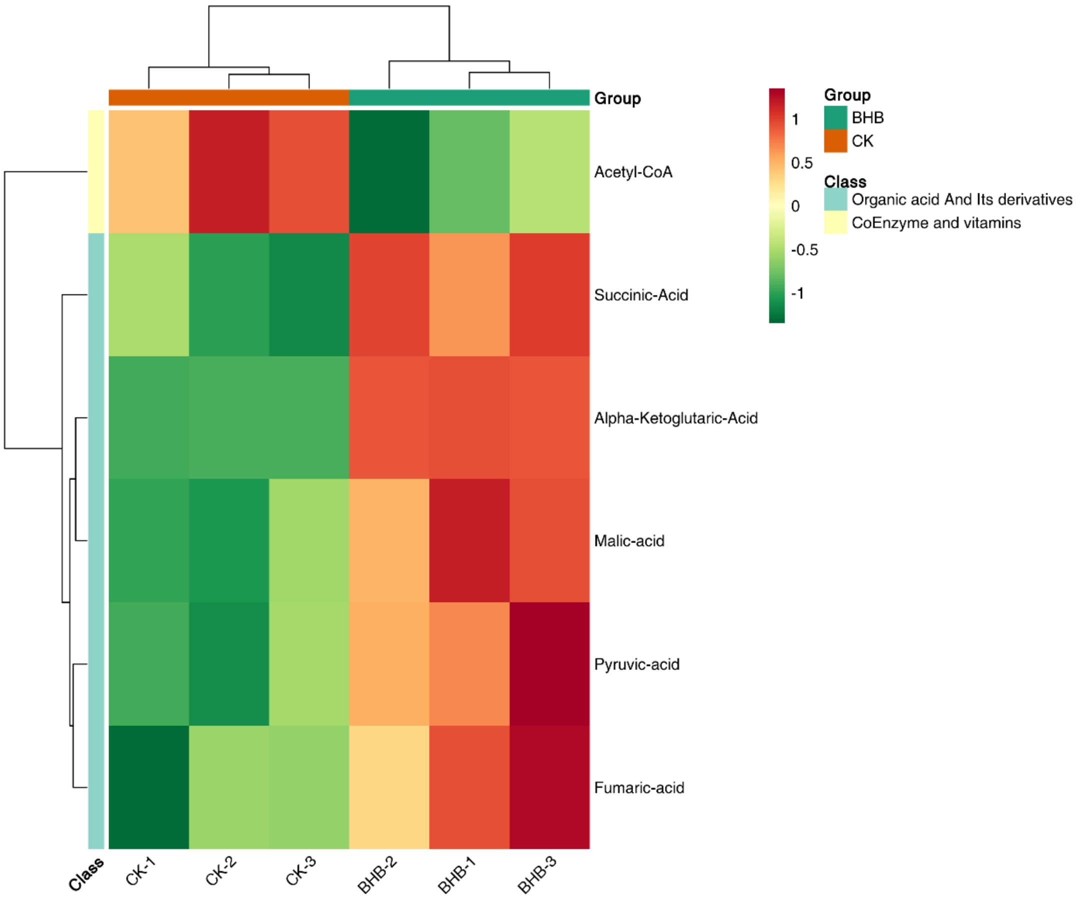

Supplementary Figure S5. Differential metabolites in the TCA cycle before and after BHB addition.
